# Update of the cooling protocol for antibiotic-free storage of boar semen at 5°C improves sperm quality and maintains low bacterial counts

**DOI:** 10.1371/journal.pone.0305280

**Published:** 2024-06-12

**Authors:** Anne-Marie Luther, Thu Quynh Nguyen, Jutta Verspohl, Dagmar Waberski

**Affiliations:** 1 Unit for Reproductive Medicine/Clinic for Pigs and Small Ruminants, University of Veterinary Medicine Hannover, Hannover, Germany; 2 Institute for Microbiology, University of Veterinary Medicine Hannover, Hannover, Germany; University of Florida, UNITED STATES

## Abstract

Preserving boar semen at 5°C instead of the conventional storage temperature of 17°C would enable a reduction of antibiotic use in pig insemination. To protect the chilling-sensitive boar spermatozoa, holding the extended semen at a higher temperature before cooling could be beneficial and facilitate the implementation of the innovative preservation concept in practice, provided that bacterial growth is kept at a low level. The aim of this study was to introduce a holding time (HT) at 17°C before cooling and to examine the effect on sperm quality and bacterial growth compared to the original cooling protocol for antibiotic-free 5°C semen storage. A series of experiments with semen doses from eight boars extended in Androstar^®^ Premium without conventional antibiotics revealed that sperm kinematics and the integrity of sperm plasma membranes and acrosomes were improved with HT between 16 and 24 h followed by delayed cooling with 0.04°C/min when compared to the original protocol for semen preservation at 5°C (p < 0.05). Both a shorter HT of 6 h and a faster cooling rate of 0.07°C/min reduced sperm quality (p < 0.05). The HT for 24 h did not compromise the inhibitory effect on bacterial growth during long-term semen storage at 5°C, not even in semen doses spiked with *Serratia marcescens*. In conclusion, semen storage at 5°C with the modified cooling protocol improved sperm quality and is antimicrobially efficient. It thus presents a ready-to-use tool for a reduction or replacement of antibiotics in pig insemination.

## Introduction

In view of the global threat of antimicrobial resistance from animal sources, the routine use of antibiotics in semen extenders is under debate. This especially applies to pig reproduction, given that circa 95% of the breeding sows are inseminated with preserved semen that is stored in a liquid state at a relatively high temperature (17 ± 1°C). To combat the risk of bacterial growth, up until now antibiotics have been consistently added to semen doses, thus favoring the generation and spreading of resistance into the environment [1]. Among the multiple tested alternative approaches [2,3], cold storage of boar semen at 5°C without antibiotics is a possible solution, inhibiting bacterial growth and yielding high fertility [4–6]. Until recently, hypothermic storage of boar semen was limited due to the species-specific high chilling sensitivity of the spermatozoa. The renewal of boar semen preservation at 5°C was finally enabled by the combination of a membrane-protective extender medium and a slow, controlled cooling protocol [4,7]. European legislation now allows semen trade without the addition of antibiotics [8], thereby motivating AI centers and pig breeders to try out the alternative method. The greatest incentive to test the 5°C storage lies in the inhibition of multi-drug resistant fast-growing bacteria with a high sperm toxicity. In case of inefficiency of conventional antibiotics, currently the cold-semen storage is the only instantly available tool in AI practice to keep resistant bacteria below spermicidal levels [9].

The suitability of the hypothermic preservation under routine semen processing conditions was recently shown across boar breeds and ages [10]. Yet, a main limitation for its implementation in AI practice is seen in the logistics industry, which worldwide is used for semen transport and storage at 17°C. Preferably, the AI centers could use their established transport logistics to the breeding farms, where the semen doses then would be stored at 5°C until use. A recent study reports that porcine commercial semen doses cooled to 5°C one day after storage at 17°C maintained high sperm functionality and low bacterial load compared to their 17°C controls, particularly in semen contaminated with *Serratia marcescens* [11]. It is as yet unclear whether this modification of semen cooling has an effect on sperm quality and bacterial growth rates compared to the original cooling protocol established for the 5°C storage [7].

Holding times of extended semen of 24 h at 17°C may increase the resistance of boar spermatozoa to cooling stress by stabilizing the membrane lipid architecture and thus would even give an advantage for the quality of sperm stored at 5°C [12]. However, in case of absence or inefficiency of antibiotics in semen extenders, there is concern regarding bacterial growth during extended holding times at the higher temperature.

Beyond this background, the aim of this study was to test cooling protocols with prolonged holding times at 17°C using an antimicrobially active semen extender, and to evaluate their impact on sperm quality and bacterial load in boar semen stored in the absence of conventional antibiotics at 5°C. The suitability of a modified cooling protocol for transfer into AI practice is elucidated.

## Material and methods

### Experimental designs

A series of five experiments were performed to evaluate the influence of different HT at 17°C and cooling rates on sperm quality (Experiments 1–4) and bacterial growth (Experiments 3 and 5) including *S*. *marcescens* used as indicator for fast growing bacteria in extended semen (Experiment 5). In Experiment 1, a HT of 18 h followed by a cooling rate of 0.07°C /min was tested. This cooling rate is received by placing a single 90 mL semen tube without insulation in a 5°C refrigerator and, therefore, was considered as rapid cooling. In Experiments 2–5, HT between 6 and 24 h were tested with a slower cooling rate, i.e. 0.04°C /min. This cooling rate was adapted from the original cooling protocol for antibiotic-free 5°C semen storage [7]. In Experiments 1, 2 and 5, the original standard cooling protocol [7] was used as control. In general, the experiments were conducted with pairwise comparison of samples to enable the use of the same cooling cabinet without interrupting the programmed cooling curves. An overview of the experimental design is shown in Table 1. In each experiment, single ejaculates of different boars were used.

**Table 1:** Overview of experimental design.

Experiment/ (Boars, n)	Sample 1 (Control) HT at 17 C	Sample 2 HT at 17°C	Sample 3 HT at 17°C	Average cooling rate from 17°C to 5°C after HT	Test set
1 (n = 9)	-^a^	18 h	-	0.07°C /min	Spermatology
2 (n = 10)	-^a^	24 h	-	0.04°C /min	Spermatology
3 (n = 6)	24 h	16 h	6 h	0.04°C /min	Spermatology Microbiology
4 (n = 8)	24 h	12 h	-	0.04°C /min	Spermatology
5.1 (n = 4)^b^ 5.2 (n = 4)^c^	-^a^	24 h	-	0.04°C /min	Microbiology *S*. *marcescens*

^a^Original cooling protocol [7]: Cooling from 30°C to 10°C with 0.04°C/min, and from 10°C to 5°C with 0.01°C/min.

^b^Bacterial concentration in the spiked semen dose: ~10^3^ CFU/mL.

^c^Bacterial concentration in the spiked semen dose: ~10^4^ CFU/mL.

HT: Holding time of the extended semen.

### Chemicals and media

Chemicals were of analytical grade and purchased from Beckman Coulter GmbH (Krefeld, Germany), Carl Roth GmbH & Co. KG (Karlsruhe, Germany), Enzo Life Sciences GmbH (Lörrach, Germany), Merck KGaA (Darmstadt, Germany), Oxoid Deutschland GmbH (Wesel, Germany), Sigma-Aldrich Productions GmbH (Steinheim, Germany), and Thermo Fisher Scientific, Inc. (Waltham, MA, USA). The semen extender was obtained from Minitüb GmbH (Tiefenbach, Germany) and was sterile filtered before the microbiology experiments.

### Semen collection, processing, and cooling

Semen was collected as entire ejaculates without the secretion of the bulbourethral glands from 10 fertile boars, 12 months to 5 years old, using the gloved-hand method. The boars of different breeds (Piétrain, Landrace, Duroc, Large White) were housed in individual pens and used for routine semen collection by trained personnel. Housing conditions and any handling of the boars were performed in accordance with the European Commission Directive for Pig Welfare and were approved by the Institutional Animal Welfare Committee of the University of Veterinary Medicine Hannover, Foundation, Hannover, Germany. Normospermic semen was extended isothermically in one step with the Androstar Premium extender without conventional antibiotics containing an organic bactericidal supplement (Minitüb GmbH; Ref. 13533/7001; [13]) to 20 × 10^6^ sperm/mL at a final volume of 100 mL. Semen doses were either cooled according to a previously established standard protocol [7] or kept for 90 min at room temperature, and then subjected to different holding times at 17°C followed by cooling at a rate of 0.04°C/min or 0.07°C min to 5°C in a programmable climate cabinet (TC200P; Minitüb GmbH; REF.: 14160/9000; 14160/0330). Briefly, in the standard protocol freshly extended semen samples were cooled from 30°C to 10°C with 0.04°C/min, and from 10°C to 5°C with 0.01°C/min [7]. After cooling, all samples were stored at 5 ± 1°C in the dark.

**Fig 1:**
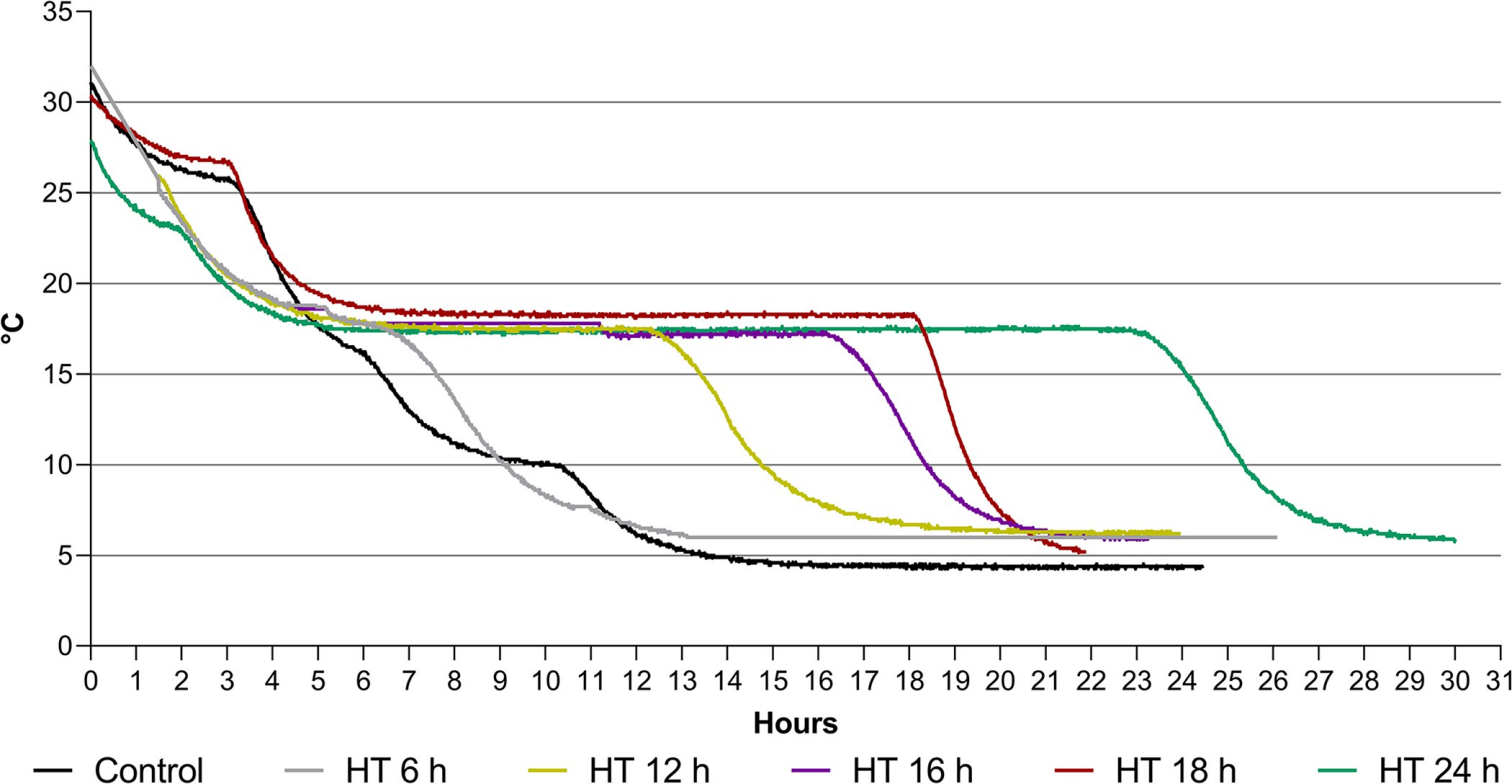
Holding times (HT) at 17°C and cooling rates from 17°C to 5°C in Experiments 1–5. Control in Experiments 1, 2 and 5: Cooling curve for semen storage at 5°C established by Paschoal et al. [7].

Temperature during semen cooling was controlled with a multiple-channel data logger (Mikromec1 Multisens MLm 424, Technetics GmbH, Freiburg, Germany) equipped with an ultra-flexible sensor positioned in the center of a tube filled with water [7]. The temperature was recorded every 1 min for 24 h. An additional sensor was placed beside the tubes to record the temperature of the climate cabinet. The respective cooling curves are presented in Fig 1.

### Spermatology

Semen was analyzed at 24 h, 48 h, 72 h, and 144 h after collection. Sperm kinematics were measured using the computer-assisted semen analysis (CASA) system AndroVision® (Version 1.2, Minitüb GmbH) as previously described [9]. Briefly, semen aliquots were incubated at 38°C for 30 min under air and then filled in a 20 μM Leja Counting Chamber (Leja Products B.V., Nieuw Vennep, The Netherlands). At least 500 spermatozoa were recorded and assigned as “motile” when their curved-line velocity was higher than 24 μm/s and the amplitude of lateral head displacement (ALH) was higher than 1 μm.

Sperm membrane integrity was assessed using the ‘Cyto Flex’ flow cytometer equipped with ‘CytExpert 2.30 Software (Beckman Coulter GmbH) [9]. Briefly, semen aliquots were stained with three fluorescent dyes, 1.0 μg/mL propidium iodide (PI), 0.6 μg/mL fluorescein conjugated peanut agglutinin (FITC-PNA), and 0.45 μg/mL Hoechst 33342 (all final concentrations). After incubation for 5 min at 38°C, 10,000 events were analyzed. A positive stain for Hoechst 33342 and negative stains for PI and FITC-PNA were indicative of spermatozoa with an intact plasma membrane and an intact acrosome. These were recorded as “membrane intact”.

### Microbiology

Bacterial counts in raw semen and semen samples stored at 5°C were determined from a 10-fold serial dilution prepared in phosphate-buffered solution (PBS) ranging from 10^−1^ to 10^−10^. From each dilution, a volume of 100 μL was plated on Columbia agar with sheep blood and incubated for 24 to 48 h at 37°C under aerobic conditions. Bacterial colonies were counted, and total bacterial numbers were calculated and recorded as colony-forming units per milliliter (CFU/mL).

Extended semen samples in Experiment 5 were spiked with *S*. *marcescens* isolated from commercial boar semen doses [9]. For this, bacteria were cultured on Columbia agar with sheep blood (Oxoid Deutschland GmbH) for 24 h at 37°C before inoculation. Bacterial colonies were added to 2 mL extender medium and bacterial concentrations were adjusted after density photometry (SDM5, Minitüb GmbH). Extended semen samples were spiked with the bacterial solutions to a final concentration of approximately 10^3^ CFU/mL (Experiment 5.1) or 10^4^ CFU/mL (Experiment 5.2). Immediately after inoculation (0 h), the bacterial count in the extended semen was determined. Samples were then cooled and stored up to 144 h in three variants: cooling to and storage at 17°C (positive control to verify the growth of *S*. *marcescens*); HT at 17°C for 24 h and then cooling to 5°C with 0.04°C/min; cooling to 5°C according to the standard protocol [7].

### Statistical analysis

Data were analyzed using IBM SPSS Statistics Professional (SPSS, IBM, Inc., Armonk, NY, United States). Data were checked for normal distribution with the Shapiro–Wilk Test. Measurements were considered significant when p < 0.05. Spermatological data are presented as mean ± standard deviation and a two-factorial ANOVA with repeated measurements or pairwise comparisons using Student’s t-test were performed. Microbiological data are presented as mean ± standard error of mean.

## Results

### Sperm kinematics

**Fig 2:**
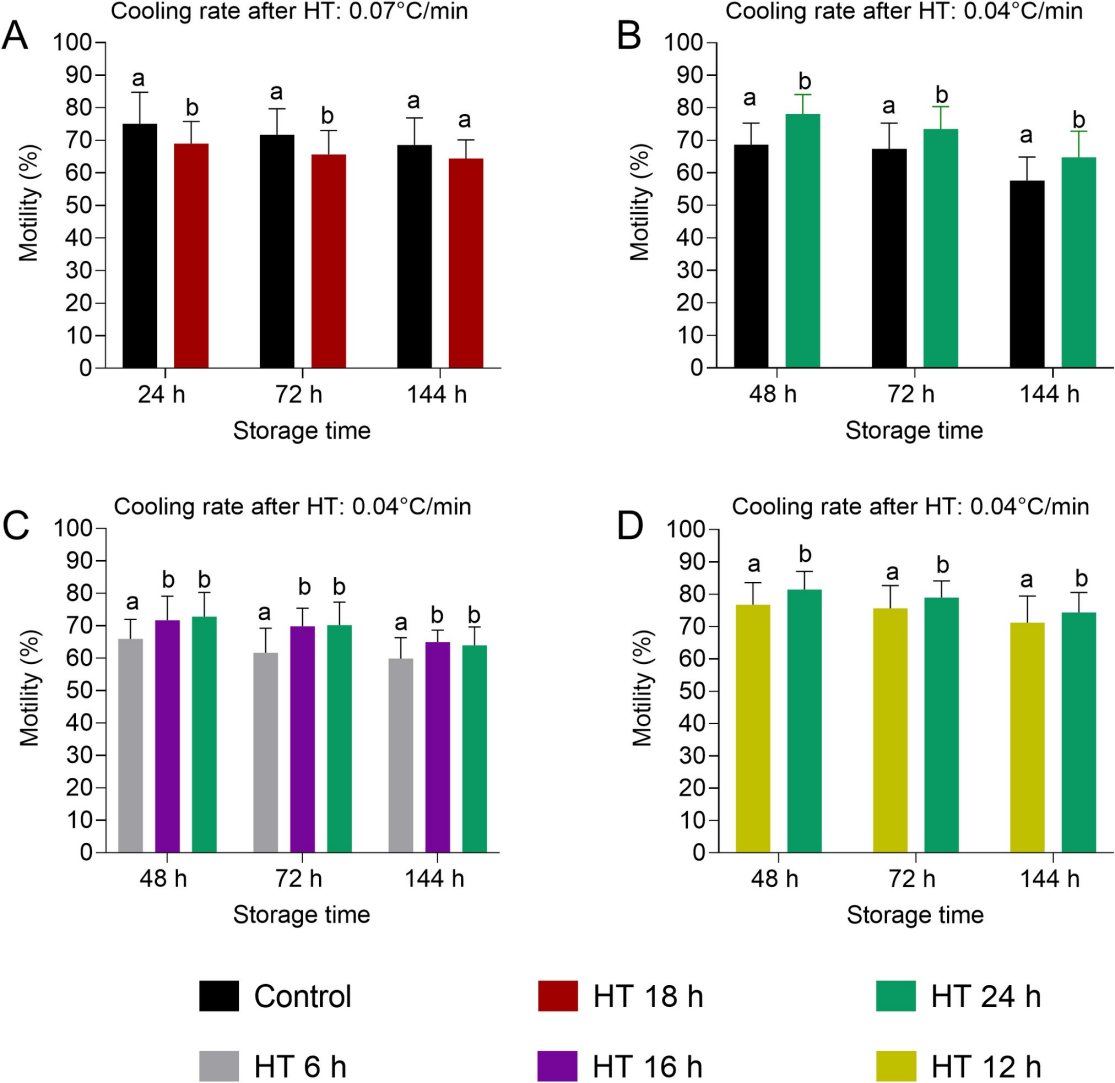
Motile spermatozoa (total, %) in extended semen samples subjected to different holding times (HT) at 17°C and subsequent cooling to 5°C. In Experiment 1 (A), the cooling rate from 17°C to 5°C was 0.07°C/min. In Experiments 2 (B), 3 (C), and 4 (D), the cooling rate was 0.04°C/min. All semen samples were then stored at 5°C up to 144 h. In Experiments 1 and 2, the previously established cooling protocol [7] served as control. Data are shown as mean and SD. a,b: Values with different superscripts differ (p < 0.05).

Sperm motility (the percentage of all motile spermatozoa) was evaluated with five different HT at 17°C and two different cooling rates to 5°C (Experiments 1–4, Fig 2). Rapid cooling (0.07°C/min) to 5°C after 18 h HT revealed lower motility until 72 h of storage compared to the original, slower cooling protocol (Experiment 1, Fig 2A). Slower cooling (0.04°C/min) after 24 h HT yielded higher motility at all storage time points compared to the control (Experiment 2, Fig 2B). Reduction of the HT to 16 h did not affect motility compared to a HT of 24 h, whereas a shorter HT of 6 h revealed lower motility (Experiment 3, Fig 2C). The HT of 12 h resulted in a small reduction of motility at all storage time points with mean differences ranging between 3.1 and 4.7% compared to a HT of 24 h (Experiment 4, Fig 2D). The short HT of 6 h resulted in reduced sperm velocity, beat cross frequency (BCF), and ALH, whereas linearity of sperm movement was not affected (Table 2).

**Table 2:** Sperm kinematics in extended semen samples subjected to different holding times (HT) at 17°C, followed by cooling to 5°C, and subsequent storage at 5°C for 144 h. In Experiment 1, the cooling rate from 17°C to 5°C was 0.07°C/min. In Experiments 2–4, the cooling rate was 0.04°C/min. In Experiments 1 and 2, the previously established cooling protocol [7] served as control. Data are shown as mean and SD. a,b: Values with different superscripts differ (p < 0.05).

	Storage time	VCL (μm/s)	Linearity	ALH (μm)	BCF (Hz)
**Experiment 1** **n = 9**	Control HT 18 h	129.8 ± 19.1^a^ 118.3 ± 9.4^a^	0.38 ± 0.05^a^ 0.38 ± 0.04^a^	1.13 ± 0.16^a^ 1.05 ± 0.08^a^	22.3 ± 2.7^a^ 20.7 ± 2.4^a^
**Experiment 2** **n = 10**	Control	81.3 ± 21.5^a^	0.38 ± 0.04^a^	0.75 ± 0.17^a^	17.0 ± 2.5^a^
	HT 24 h	86.1 ± 21.5^a^	0.36 ± 0.04^a^	0.80 ± 0.17^a^	18.9 ± 2.7^b^
**Experiment 3** **n = 6**	HT 6 h	104.8 ± 8.5^a^	0.35 ± 0.03^a^	0.95 ± 0.07^a^	17.8 ± 2.1^a^
	HT 16 h	115.2 ± 6.3^b^	0.35 ± 0.02^a^	1.02 ± 0.05^b^	19.3 ± 1.6^b^
	HT 24 h	111.6 ± 10.8^b^	0.35 ± 0.02^a^	1.00 ± 0.08^b^	18.7± 2.0^ab^
**Experiment 4** **n = 8**	HT 12 h	135.3 ± 15.0^a^	0.29 ± 0.03^a^	1.20 ± 0.12^a^	19.6 ± 2.7^a^
	HT 24 h	142.5 ± 11.4^b^	0.30 ± 0.03^b^	1.25 ± 0.08^a^	20.5 ± 2.2^a^

VCL: Curved-line velocity; ALH: Amplitude of lateral head displacement; BCF: Beat cross frequency.

### Sperm membrane integrity

**Fig 3:**
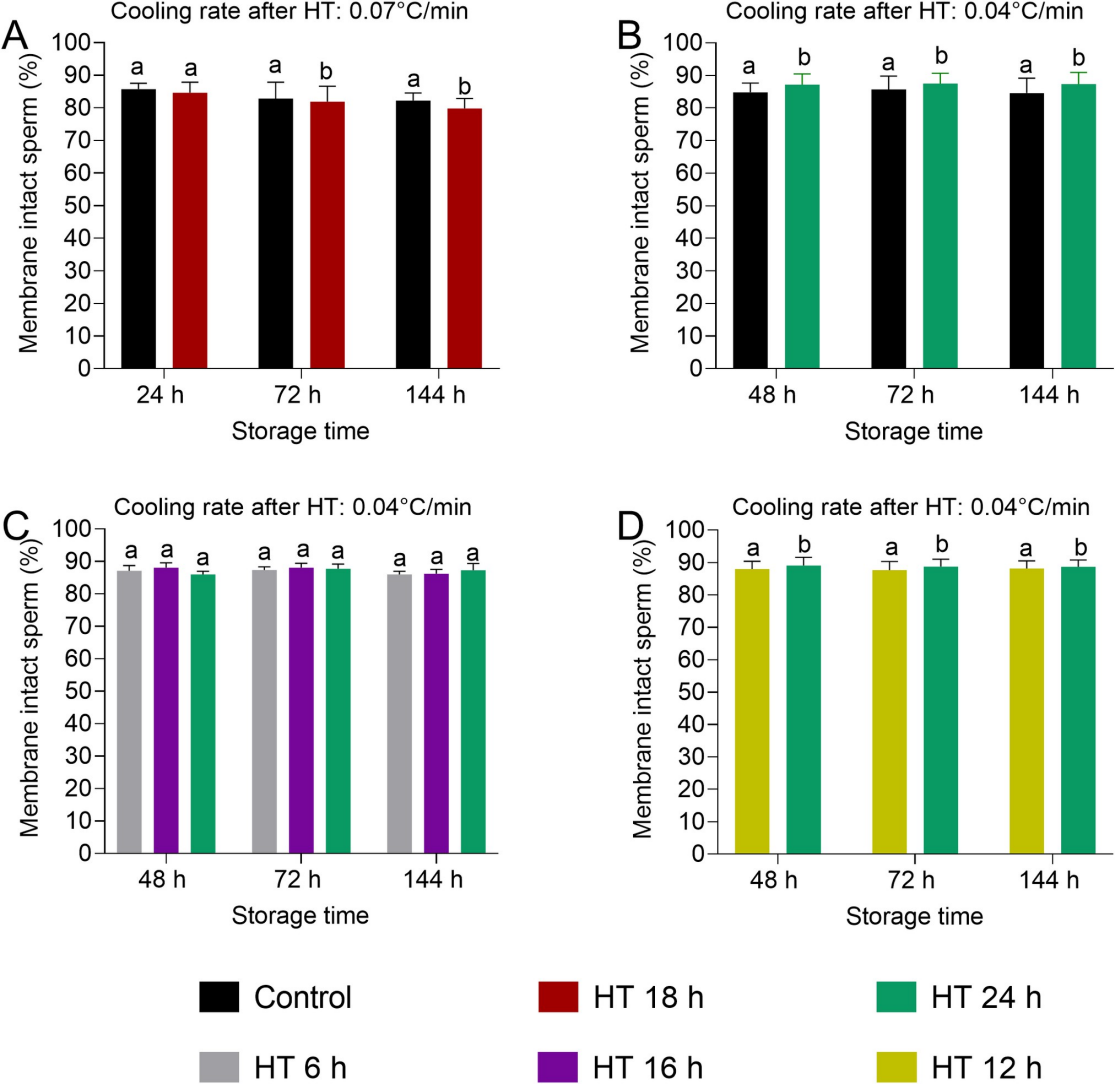
Spermatozoa with intact plasma membranes and acrosomes (%) in extended semen samples were subjected to different holding times (HT) at 17°C and subsequent cooling to 5°C. In Experiment 1 (A), the cooling rate from 17°C to 5°C was 0.07°C/min. In Experiments 2 (B), 3 (C), and 4 (D), the cooling rate was 0.04°C/min. All semen samples were then stored at 5°C up to 144 h. In Experiments 1 and 2, the previously established cooling protocol [7] served as control. Data are shown as mean and SD. a,b: Values with different superscripts differ (p < 0.05).

Sperm membrane integrity was evaluated with five different HT at 17°C and two different cooling rates to 5°C (Experiments 1–4, Fig 3). Rapid cooling (0.07°C/min) to 5°C after 18 h HT resulted in a small reduction of sperm membrane integrity at 72 h and 144 h of storage compared to the original, slower cooling protocol (Experiment 1, Fig 3A). Slower cooling (0.04°C/min) after 24 h HT yielded higher membrane integrity at all storage time points with mean differences ranging between 1.9 and 2.7% compared to the control (Experiment 2, Fig 3B). There was no or a small difference between HT 6 h, 12 h, 16 h, and 24 h prior to slow cooling to 5°C (Experiments 3 and 4, Fig 3C and 3D).

### Microbiology

**Fig 4:**
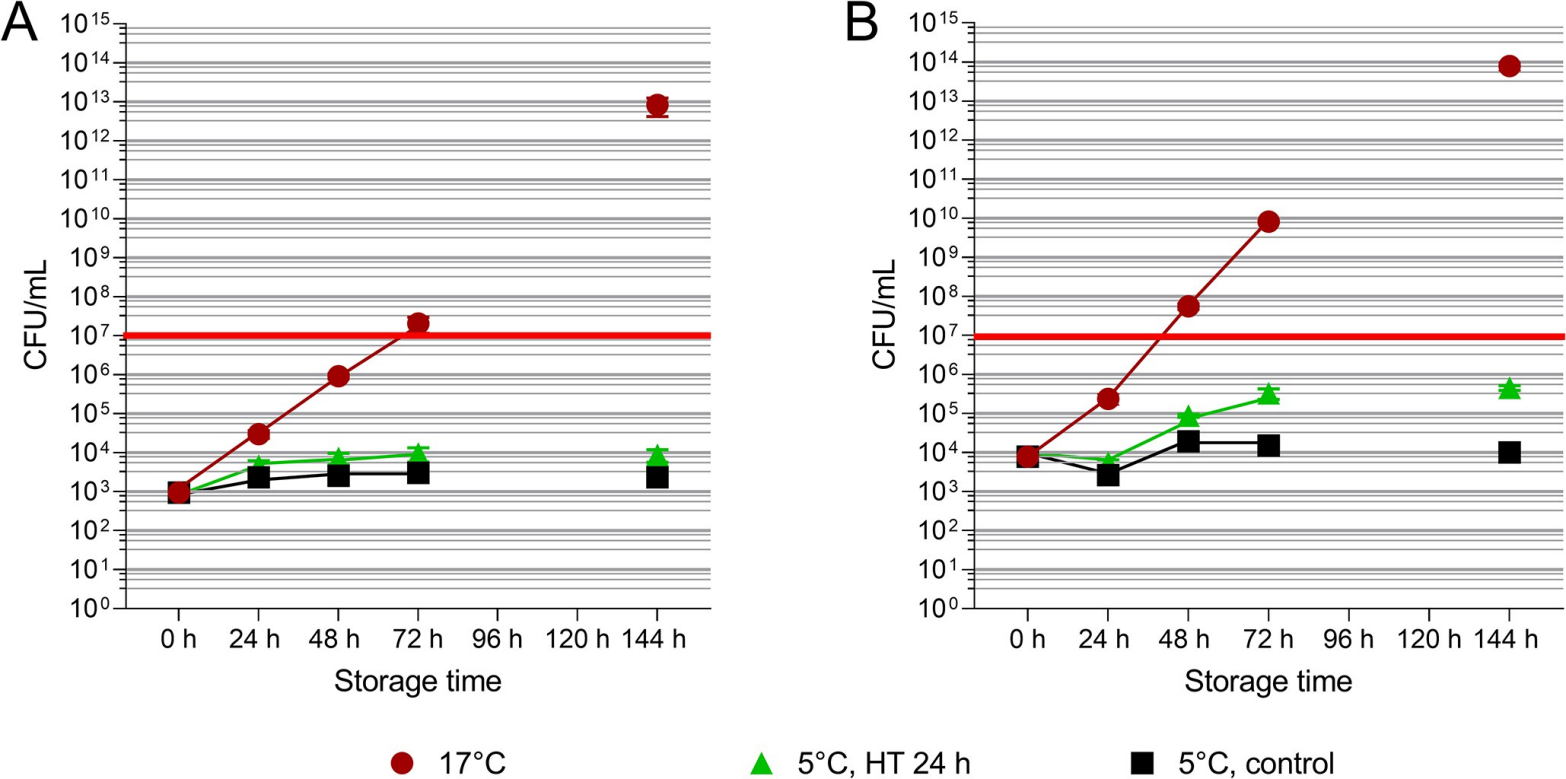
Bacterial growth (CFU/mL) in extended semen samples spiked with *S*. *marcescens* to ~ 10^3^ CFU/mL (Experiment 5.1 (A)) or ~ 10^4^ CFU/mL (Experiment 5.2 (B)). Samples with a holding time (HT) of 24 h at 17°C and subsequent cooling to 5°C were compared to the previously established cooling protocol [7] as control. Additional semen samples were stored at 17°C as positive control for bacterial growth. The red line marks the threshold for spermicidal concentrations of *S*. *marcescens* [14]. Data are shown as mean and SEM of four semen samples from different boars in each experiment.

Bacterial counts were determined in semen samples with their original bacterial load in raw semen and in extended semen samples. After 144 h of storage, bacterial counts were below the detection limit (< 10 CFU/mL), regardless of the HT duration (Experiment 3, Table 3). In samples spiked with *S*. *marcescens* to a concentration ~ 10^3^ CFU/mL in the semen dose, bacterial counts remained on a low level (~ 10^4^ CFU/mL) during semen storage at 5°C for 144 h, whereas the samples stored at 17°C (positive control) showed exponential growth up to 10^13^ CFU/mL (Experiment 5.1, Fig 4A). A higher initial bacteria load (~ 10^4^ CFU/mL) in semen samples spiked with *S*. *marcescens* showed a moderate increase to ~ 5 × 10^5^ CFU/mL after 144 h storage at 5°C, and remained below spermicidal levels during the storage period (Experiment 5.2, Fig 4B).

**Table 3:** Bacterial counts (CFU/mL) in semen doses after different holding times (HT) at 17°C, followed by cooling to 5°C at 0.04°C/min, and storage for 144 h (Experiment 3).

	Raw semen	Extended semen		
	**0 h**	**HT 6 h**	**HT 16 h**	**HT 24 h**
**CFU/mL**	9.6 x 10^3^ ± 1.0 x 10^4^	< 10	< 10	< 10

## Discussion

The present study shows that holding times of between 16 h and 24 h at 17°C of freshly processed boar semen improved sperm quality compared to the original protocol established for cold storage [7]. Importantly, extended holding times did not compromise the inhibitory effect on bacterial growth, so that the concept of antibiotic-free semen storage at 5°C remains valid. Thus, the modified protocol has two advantages: first, sperm quality was improved and second, the implementation of the novel preservation in AI practice was facilitated. Artificial insemination centers can proceed with the conventional semen processing chain including transport and delivery of the semen to the sow farms within one day after production. Farmers receiving the semen already on the day of collection could use it the same day without compromising biosecurity, and then, ideally within a time frame of 16 to 24 h after semen processing, store the remaining semen doses at 5°C. Alternatively, the semen could be hold at 17°C and cooled to 5°C in the AI center followed by shipping at 5°C the next day. This could have additional benefit for sperm quality, especially for long distance shipping, because semen transported at 5°C is less sensitive to vibration stress compared to 17°C [15,16].

Storage of extended boar semen for one day at 17°C in the absence of antibiotics is a safe procedure [17], especially if, like in the present study, semen extenders with an intrinsic antimicrobial activity are used. Recently, it was shown that the long-term extender Androstar Plus without conventional antibiotics containing an organic bactericidal supplement (OBS) maintained bacterial load below spermicidal levels [18]. Although the role of OBS in the antimicrobial activity is unclear, it is noted that the Androstar Premium used in the present study, in addition to membrane protective components, also contained OBS [13]. The use of antimicrobial effective semen extenders during the HT at 17°C may be crucial for biosecurity when using the modified cold storage protocol.

In the present study, microbial safety was verified not only for regular semen doses prepared from raw semen with a typical load of commensal bacteria around 10^4^ CFU/mL [19], but also in semen spiked with two doses of *S*. *marcescens*. This bacterial species has the fastest growth rate among the critical, highly sperm-toxic bacteria [14] and was therefore used as an indicator to identify the risk of bacterial growth during delayed cooling. The inhibitory effect of cold storage shown in our previous study [9] and confirmed here, was not compromised by holding semen for one day at 17°C and subsequent slow cooling to 5°C. Cold storage limited bacterial counts to values (10^4^–10^5^ CFU/mL), which do not affect sperm functionality [9,14], whereas, as expected, spermicidal levels of > 10^7^ CFU /mL [20] were already reached between 48 h and 72 h in the 17°C controls.

The holding time was adapted from classical cryopreservation protocols based on the recognition that delayed cooling stabilizes the lipid architecture of the sperm´s plasma membrane and thus renders spermatozoa less sensitive to subsequent cooling [21,22]. Unlike in typical cryopreservation protocols, where the sperm-rich fraction is hold 1:1 diluted in a semen extender, hypothermic liquid stored spermatozoa are not exposed to high concentrations of seminal plasma during the HT. This supports the view that the HT is important, whereas the additional benefit from prolonged contact to seminal plasma [23,24] is less relevant for the sperm to acquire resistance to cooling [21]. Major stress on membranes occurs during phase changes in lipids in the vicinity of 15°C– 5°C [25], which is mitigated by slow cooling at a rate of 0.08 to 1.5°C /min [24]. The present study confirms the importance of slow cooling in this temperature range for the liquid cold storage. The faster cooling rate of 0.07°C/min, which corresponds to placing a single 90 mL semen tube in a refrigerator at 5°C, impaired sperm kinematics and membrane integrity compared to the original protocol with a cooling rate of 0.02°C/min [7]. Hence, in association with storage time, the cooling rate of 0.04°C/min between 17°C and 5°C was appropriate. Storing semen doses in bundles in the refrigerator is commonplace in AI practice, thus creating insulation and enabling delayed cooling. Nonetheless, it is recommended to adapt the semen storage in the individual farm situation to a cooling rate between 0.02 and 0.04°C/min. Slower cooling should be avoided to ensure that the semen reaches the antimicrobial safe temperature of 5°C in time. Starting from semen dilution, reaching the intended storage temperature at 5°C was delayed by up to 9 h in the updated protocol with HT compared to the original protocol (i.e., from 18 h to 29 h). Obviously, the longer exposure above 5°C does not increase bacterial replication in the extended semen. This seems logical because 17°C is below the optimum growth temperature of around 30°C for mesophilic bacteria, which the opportunistic pathogens identified in boar semen typically belong to [26].

## Conclusion

The present study provides an update for the original cooling protocol designed for the storage of boar semen at 5°C. The beneficial effect of holding the extended semen at 17°C between 16 h and 24 h resides in the improved sperm quality, and additionally, the implementation into AI practice is facilitated. The time extension above 5°C does not compromise the antimicrobial effect, and thus maintains biosafety in the absence of conventional antibiotics. Besides optimizing semen processing and cooling, the choice of a cold-shock protecting semen extender with intrinsic antimicrobial activity is crucial. Low-temperature semen storage has now become a ready-to-use technology in pig AI that contributes to sustainable pig reproduction in the sense of the One Health approach. It may become indispensable if effective antibiotics in semen extenders are no longer available, especially as long as alternate antimicrobial concepts are not in practical use.

## Supporting information

S1 Data This table shows the original data of all experiments in this study. (XLSX)
